# Supplementary material for: Efficacy of Ursolic Acid-Enriched Water-Soluble and Not Cytotoxic Nanoparticles against Enterococci
Source: Pharmaceutics. 2021 Nov 21;13(11):1976. doi: 10.3390/pharmaceutics13111976 (PMC8625572; doi:10.3390/pharmaceutics13111976)
Supplement: Supplementary file 1 [file pharmaceutics-13-01976-s001.zip › pharmaceutics-1469579-supplementary.pdf]

pharmaceutics

Supplementary Materials:Efficacy of Ursolic Acid-Enriched Water-Soluble and Not Cytotoxic Nanoparticles against Enterococci

Anna Maria Schito, Debora Caviglia, Gabriella Piatti, Alessia Zorzoli, Danilo Marimpietri, Guendalina Zuccari, Gian Carlo Schito and Silvana Alfei

Section S1. Synthesis and Characterization of UA-loaded dendrimer nanoparticles (UA-G4K NPs) [1].

S1.1. Preparation of UA-Loaded Dendrimer NPs (UA-G4K NPs) by Nanoprecipitation Process

The UA-G4K NPs were prepared by the nanoprecipitation technique according to Scheme S1.

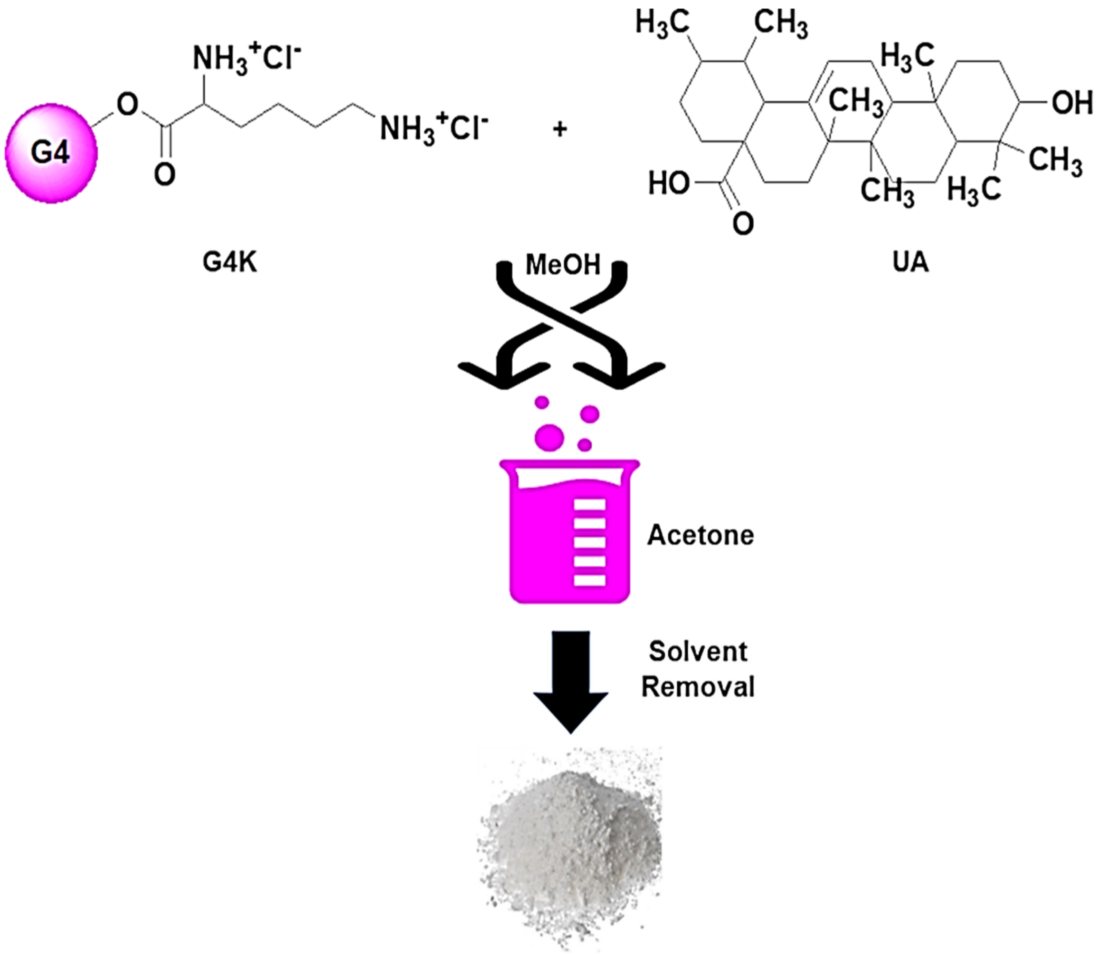

Scheme 1. Synthetic pathway to prepare UA-G4K NPs. G4 = fourth generation; K = lysine; UA = ursolic acid.

Two miscible phases, both made of organic solvents were prepared. MeOH (7.7 mL) was used to solubilize the dendrimer G4K (66.6 mg, 0.00444 mmol) and UA (88.8 mg, 0.1944 mmol). Acetone (17 mL) was used as non-solvent. The clear solution of the two ingredients (G4K and UA) was added to the acetonic phase, drop-wise using a Pasteur, at room temperature and under moderate magnetic stirring (500 rpm), obtaining a milky suspension. The evaporation of the organic solvent was performed subsequently using a Rotavapor® R-3000 (Büchi Labortechnik, Flawil, St. Gallen, Switzerland), at 70 °C and

reduced pressure. The solid residue was dissolved twice in the minimum volume of MeOH obtaining clear solutions which were precipitated in acetone, to leave in solution the eventual UA not encapsulated. After centrifugations at 3500 rpm the UA-loaded dendrimer NPs (UA-G4K NPs) purified by free UA were obtained as a pale yellow, hygroscopic glassy solid which was stored under vacuum in a dryer (105.7 mg). Not encapsulated UA was recovered by evaporating the acetonic supernatant solutions and recrystallizing the solid residue (from MeOH, off-white crystals, 35.8 mg).

FTIR (ν, cm^{-1}): 3500-3000 (NH_3^+ dendrimer), 3500-2500 (OH stretching UA), 1735 (C=O stretching esters of dendrimer), 1688 (C=O stretching carboxyl of UA), 1215, 1044 (C-O stretching esters of dendrimer).

^1H NMR (400 MHz, CD_3OD, δ, ppm): 0.75–0.98 [several s, 726 H, seven CH_3 and H (C(5)) of UA (CH_3 *core* not detected)], 1.00–2.40 [m, 1116 H (135H, CH_3 G1, G2, G3 and G4 of dendrimer + 288H, $CH_2CH_2CH_2$ lys overlapped by 594 H, CH_2 of UA + 99H, CH of UA)], 2.95–3.16 [m, 129 H (96H $CH_2NH_3^+$ lys overlapping 33H, CH of UA), 4.10–4.30 [m, 234 H (186H, CH_2O of dendrimer + 48H $CHNH_3^+$ lys), 4.58–4.70 (m, 33H, CH of UA), 5.22 (m, 33H, CH of UA), NH_3^+ lys (288H) and OH of UA (66H) were not detectable because the protons from these groups get exchanged with the proton of CD_3OD. Anal. Cald. for $C_{1508}H_{2628}N_{96}O_{285}Cl_{96}$: C, 60.24; H, 8.81; N, 4.47; Cl, 11.32%. Found: C, 60.64; H, 8.49; N, 4.96; Cl, 11.0.

S1.2. Morphology of G4K and UA-G4K Particles

The morphology of G4K (Figure S1a) and UA-G4K (Figure S1b) was investigated by scanning electron microscopy (SEM). Briefly the samples were fixed on aluminum pin stubs and sputter-coated with a gold layer (30 mA for 1 min) and were examined at an accelerating voltage of 20 kV. The micrographs were recorded digitally using the DISS 5 digital image acquisition system (Point Electronic GmbH, Halle, Germany).

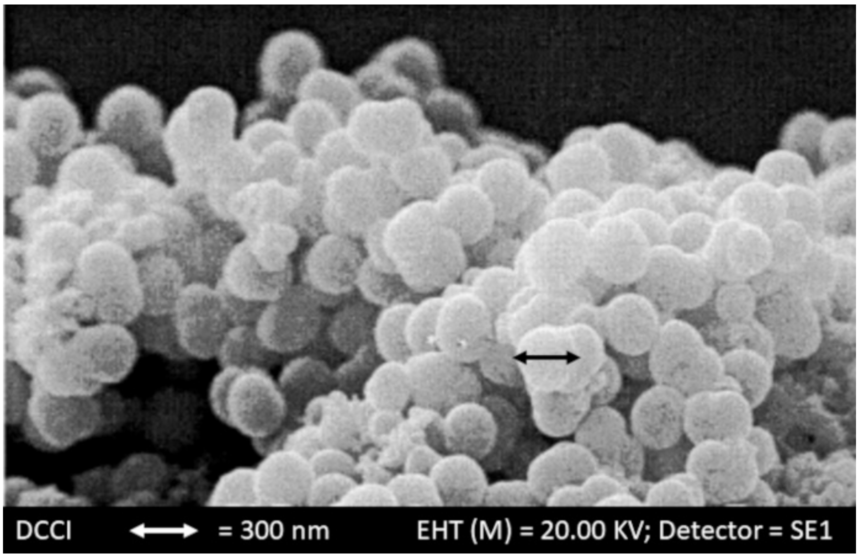

(**a**)

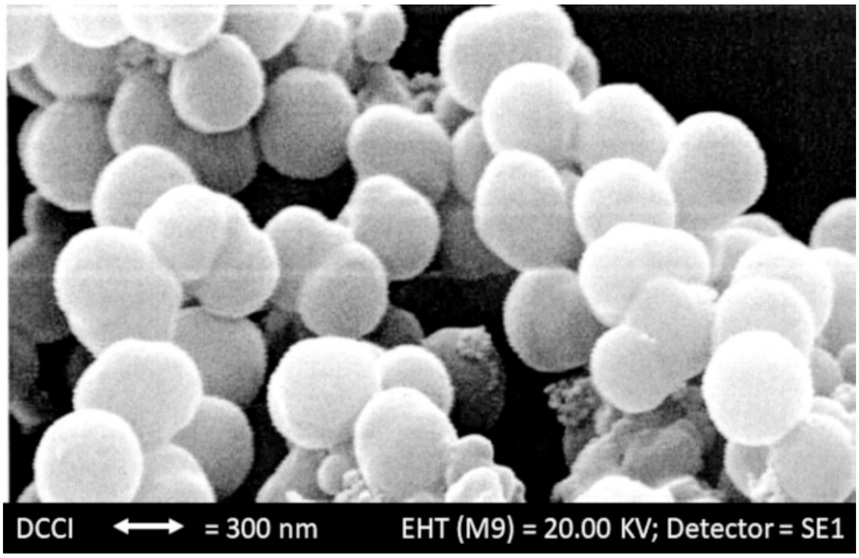

(**b**)

Figure S1. SEM images of G4K (**a**) and UA-G4K (**b**) particles.

S1.3. Content of UA in UA-G4K, Drug Loading (DL%) and Encapsulation Efficiency (EE%)

S1.3.1. UA Standard Calibration Curve

A stock solution of UA (1 mg/mL) was prepared in MeOH, and dilutions with MeOH were made to prepare standard solutions at concentrations of 50, 100, 200, 300 and 500 μg/mL. Aliquots of 20 μL were picked up from each solution and were used to construct the UA standard calibration curve. Briefly, UA in each solution was quantified using a HPLC JASCO system (Jasco Inc., Easton, MD, USA) equipped with a JASCO PU-980 pump, a JASCO UV-970-975 UV/Vis detector and an ODS C18 column (250 × 4.6 mm, 5 μm), and detecting the absorbance at λ_{max} = 210 nm. The mobile phase consisted of MeOH and 0.03 mol/L phosphate buffer (pH 2.8) with a ratio of 88:12. Determinations were made in triplicate and the peaks areas (associated to the values of absorbance measured by the UV/Vis detector) obtained for each UA concentration analysed, were expressed as mean ± standard deviation (A_{mean} ± SD). A_{mean} and C_{UA} (μg/mL) data were used to work out the UA calibration model by least squares (LS) method whose equation was Eq. (1).

$$y = 0.1757x + 0.106 \quad (1)$$

where y is the peak area (A) associated to the absorbance measured at λ = 210 nm and x is the UA concentration (C_{UA}) (μg/mL).

S1.3.2. Estimation of UA content in UA-G4K NPs

A known quantity of UA-G4K (3 mg) was dissolved in MeOH (9 mL), obtaining a final concentration of 333 μg/mL. The clear solution was vigorously stirred for ten minutes to promote the release of UA. The amount of UA in the sample was quantified at 210 nm by HPLC analysis, using the same apparatus and the same conditions described in the previous section. Particularly, six aliquots (20 μL) of the solution were analysed against a blank solution of the empty dendrimer.

The drug loading (DL%) and encapsulation efficiency (EE%) of UA-G4K were calculated from the following formulas.

$$DL\ (\%) = \frac{\text{weight of the drug in NPs}}{\text{weight of the NPs}} \times 100\%$$

$$EE\ (\%) = \frac{\text{weight of the drug in NPs}}{\text{initial amount of drug}} \times 100\%$$

Table S1 collects the results concerning the above-mentioned determinations.

Table S1. Values of peak area obtained for the six aliquots and the related C_{UA} obtained from Eq. (1), results concerning the concentration of UA in UA-G4K NPs and MW of UA-G4K, as well as the difference expressed as error % between the MW obtained by ^{1}H NMR and that computed using HPLC results.

Peak Areas	C_{UA} (μg/mL)	UA in 0.333 mg	Moles UA per mole G4K	MW	Error (%)
29.38	166.6	0.1654 ± 0.00195 mg	32.4 ± 3.8		
28.69	162.7	**UA in UA-G4K (105.7 mg)**	**DL%**		
29.01	164.5	52.5 ± 6.2 mg	49.7 ± 5.9	29804.5 ± 1735.5 [1]<br>30069.0 [2]	0.76 [3]
29.51	167.4	**MW UA, MW G4K**	**EE%**		
28.91	163.9	456.7, 14997.9	59.1 ± 5.9		
29.49	167.2				

[1] Computed considering the DL% value (HPLC analysis); [2] by ^{1}H NMR spectrum; [3] computed on the mean.

S1.4. UA-G4K Molecular Weight (MW)

The MW of UA-G4K complex was estimated both by its ^{1}H NMR spectrum and by the results of HPLC analyses, obtaining findings with a minimal difference (0.76%). In addition, the MW of UA-G4K was furtherly confirmed by elemental analysis (Table S1).

S1.5. Water Solubility of UA in the form of UA-G4K NPs and of the Nanotechnologically-Manipulated UA released in Water

The water solubility of UA-G4K and of nanotechnologically-manipulated UA released in water were determined according to a previously reported and approved procedure [2,3]. An exactly weighted amount of UA-G4K (6.1 mg) was added with successive aliquots of water m-Q (50 μL each one) and maintained under vigorous stirring at room temperature for ten minutes observing abundant foaming. The initial suspensions increasingly clarified becoming a practically limpid yellowish stable solution with no presence of aggregates after the adding of 600 μL of water. A drop of the obtained solution was observed with a Leica Galen III Professional Microscopes (Taylor Scientific, St. Louis, MO, USA) without observing precipitate or differences with a drop of pure water. Anyway, the solution was filtered using a Merck Millipore (Fisher Scientific GmbH, Schwerte, Germany) SLHN033NB filter (33 mm, 0.45 μm). The filtrate was diluted 1:20 thus having a final volume of 12 mL with MeOH and 20 μL aliquots were analyzed by HPLC using the same apparatus and the same conditions described in Section 2.6.1. The exact amount of UA which was solubilized was quantified at 210 nm using the previously constructed standard calibration curve. The determinations were made in triplicate and the UA water solubility was reported as mean ± SD (Figure S2).

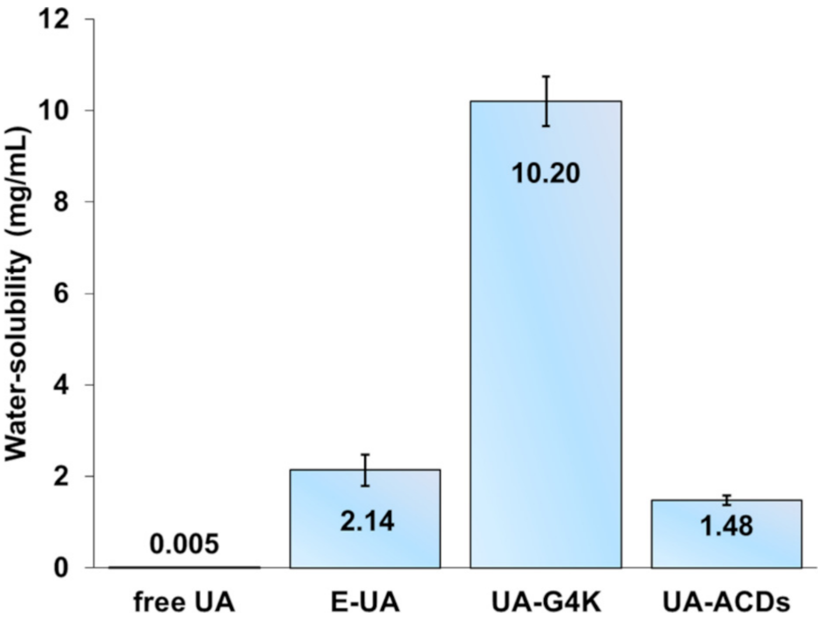

Figure S2. Water solubility of pristine UA (free UA), of nanotechnologically-manipulated UA released in water solution (HPLC) (E-UA), of UA-G4K, and of UA cyclodextrins inclusion complexes (UA-ACDs), herein reported as a mean of literature data ± SD [4].

S1.6. Dynamic Light Scattering (DLS) Analysis

Particle size (in nm), polydispersity index (PDI) and zeta potential (ζ-p) (mV) of UA-G4K were measured at 25 °C, at a scattering angle of 90° in m-Q water by using a Malvern Nano ZS90 light scattering apparatus (Malvern Instruments Ltd., Worcestershire, UK).

Solutions of UA-G4K in m-Q water were diluted to final concentrations to have 250-600 kcps. ζ-p value of UA-G4K was recorded with the same apparatus. The results from these experiments were presented as the mean of three different determinations ± SD. Concerning the particle size distribution, intensity-based results were reported. Table S2 collects the results obtained from DLS analyses on G4K and UA-G4K concerning their size (Z-ave, nm), polydispersity index (PDI) and Zeta potential (ζ-p).

Table S2. Results obtained from DLS analyses on G4K and UA-G4K: particle size (Z-ave, nm), polydispersity index (PDI) and Zeta potential (ζ-p).

Physical characteristics	G4K	UA-G4K
Z-Ave (nm)	333.4 ± 24.6	577.5 ± 10.7
PDI	0.286 ± 0.040	0.235 ± 0.028
ζ-p (mV)	+66.1 ± 4.7	−42.6 ± 4.39

Z-Ave = hydrodynamic diameter; ζ-p = Zeta potential.

S1.7. In Vitro UA Release Profile From UA-G4K NPs

In *vitro* release of UA from UA-G4K NPs was investigated using the dialysis bag diffusion technique. An exactly weighted amount of UA-G4K (10 mg) was dissolved in 1 mL of 0.1 M phosphate-buffered saline (PBS, pH = 7.4), which should assure the dissolution of the complex. The solution was then placed into a pre-swelled T2 tubular cellulose dialysis bag (flat width = 10 mm, wall thickness = 28 µm, V/cm = 0.32 mL) with a nominal molecular weight cut off (MWCO) of 6000–8000 Da (Membrane Filtration Products, Inc., Seguin, TX, USA) and immersed into 20 mL of 0.1 M PBS, pH 7.4, at 37 °C with gentle stirring for 24 h. At predetermined time intervals (1 h, 2 h, 3 h, 4 h, 5 h, 6 h, 8 h, 10 h, 12 h, 24 h), 1 mL was withdrawn from the incubation medium and was analyzed by HPLC using the same apparatus and the same conditions described in Section 2.6.1 to determine

the UA concentration. The exact amount of UA present in the samples was quantified at 210 nm, determinations were made in triplicate and reported as the mean ± SD. After sampling, equal volume of fresh PBS was immediately replaced into the incubation medium.

The concentration of UA released from UA-G4K NPs was expressed as a cumulative release percentage (%) of the total amount of UA present in the UA-G4K NPs (according to the DL% value). The UA cumulative release (%) were plotted as a function of time obtaining the curve of UA release profile (Figure S3).

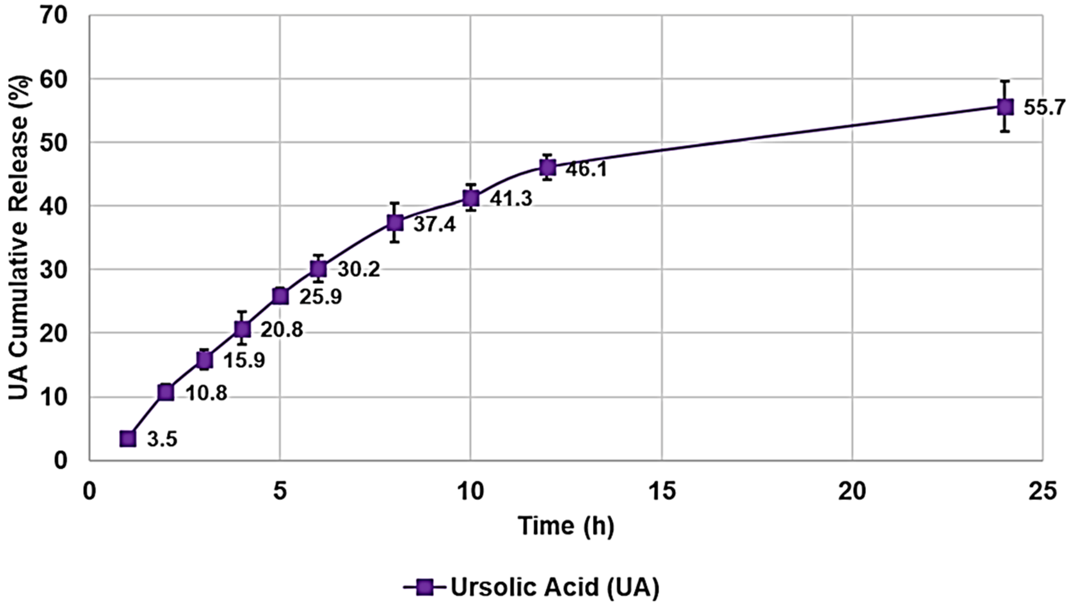

Figure S3. UA cumulative release (%) at pH 7.4 monitored for 24 hours.

To determine the kinetics and the main mechanism which govern the release of UA from UA-G4K NPs, the UA release profile was analysed by fitting the cumulative drug release curve with zero order, first order, Higuchi, Hixson-crowell and Korsmeyer-peppas mathematical models. The highest value of the coefficient of determination (R^2) of the linear mathematical models, which explains how the model is good to explain the variability of data, was considered as parameter to determine which model better fits the release data. R^2 values were 0.8202 (zero order), 0.8924 (first order), 0.9211 (Korsmeyer-peppas model), 0.8702 (Hixson Crowell model) and 0.9507 (Higuchi model) thus establishing the UA cumulative release (%) dispersion graph best fitted with Higuchi kinetic model (Figure S4).

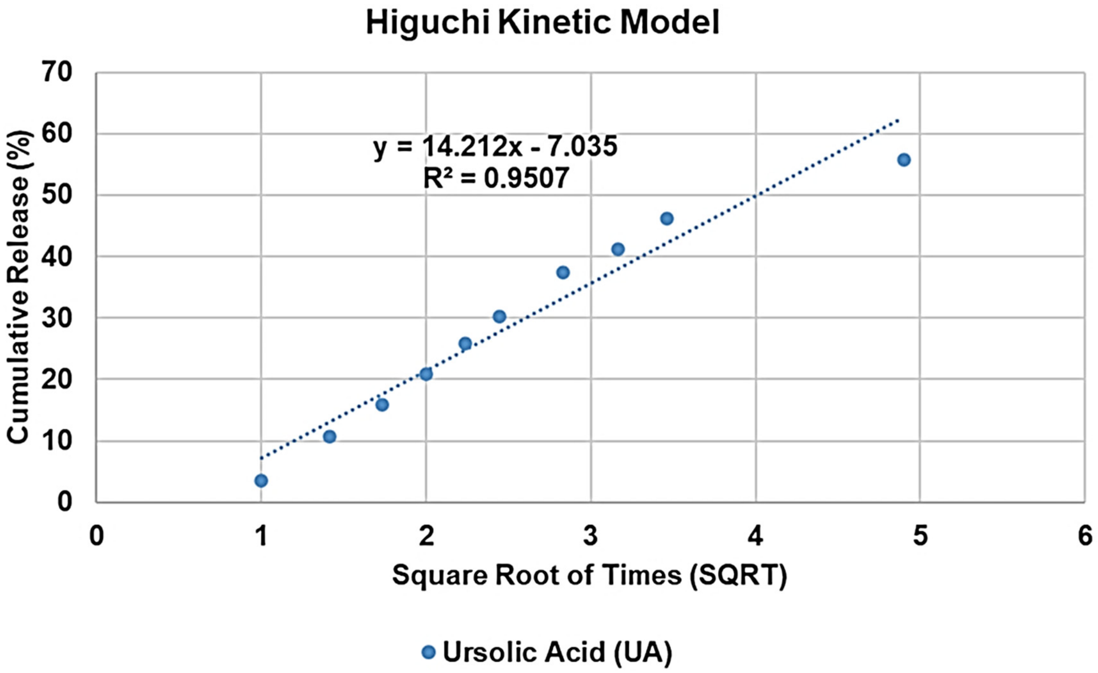

Figure S4. Linear regressions of the Higuchi kinetic mathematical model with the related equation and R^2 value.

Section S2. Biological Investigations [1].

S2.1. In Vitro Evaluation of G4K, UA and UA-G4K Cytotoxicity on HeLa Cells

The cytotoxicity of G4K, UA and UA-G4K was evaluated *in vitro* on HeLa cells purchased by Termofischer Scientific (Rodano, Milan, Italy). Briefly HeLa cells were increased in Dulbecco's Modified Eagle Medium (DMEM) enriched with Fetal Bovine Serum (FBS, 10%), non-essential amino acids (1%) and antibiotics (1%, penicillin and streptomycin) and maintained in atmosphere containing 5% CO_2 at 37 °C. The cells were seeded at the density of 2 x 10^4 cells per well in a 24-well plate and in 4-wells slides in 500 µL of medium and incubated at 37 °C for 72 h. Subsequently, the cells were incubated with increasing concentrations (5–20 µM) of G4K, UA and UA-G4K at 37°C for 24h. Then 10 µL MTT (3-(4,5-Dimethylthiazol-2-yl)-2,5-diphenyl-2H-tetrazolium bromide) was added into each well and after 4 h, the medium and MTT were discarded and 100 µL dimethyl sulfoxide (DMSO) was added into each well. Finally, optical density at 490 nm was measured on a Termofischer Scientific microplate reader (Rodano, Milan, Italy) to determine cells viability (%) Paclitaxel was essayed in the same condition as positive control. Determinations were made in triplicate and results were expressed as mean percentage of the control (untreated cells) ± standard deviation (SD) (Figure S5).

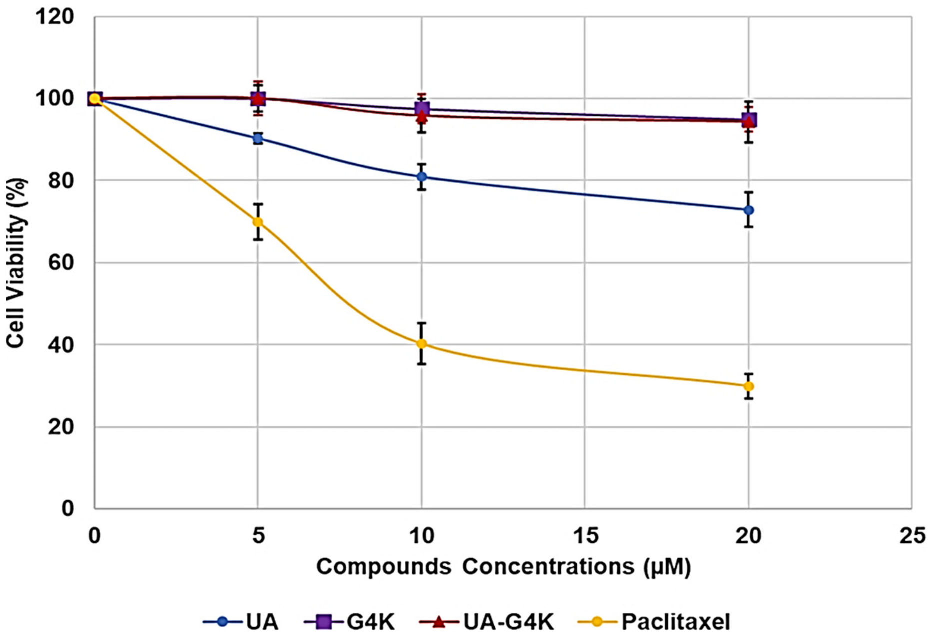

Figure S5. Cells viability of Hela cells exposed for 24 h to UA, G4K, UA-G4K and Paclitaxel at concentration 5–20 µM.

References

1. Alfei, S.; G.; Zuccari, G.; Schito, A.M. Considerable Improvement of Ursolic Acid Water Solubility by its En-capsulation in Dendrimer Nanoparticles: Design, Synthesis and Physicochemical Characterization. *Nanomaterials* **2021**, *11 (9)*, 2196.
2. Alfei, S.; Marengo, B.; Domenicotti, C. Polyester-Based Dendrimer Nanoparticles Combined with Etoposide Have an Improved Cytotoxic and Pro-Oxidant Effect on Human Neuroblastoma Cells. *Antioxidants* **2020**, *9*, doi:10.3390/antiox9010050.
3. Alfei, S.; Turrini, F.; Catena, S.; Zunin, P.; Parodi, B.; Zuccari, G.; Pittaluga, A.M.; Boggia, R. Preparation of Ellagic Acid Micro and Nano Formulations with Amazingly Increased Water Solubility by Its Entrapment in Pectin or Non-PAMAM Dendrimers Suitable for Clinical Applications. *New J. Chem.* **2019**, *43*, 2438–2448, doi:10.1039/C8NJ05657A.
4. Song, S.; Gao, K.; Niu, R.; Yi, W.; Zhang, J.; Gao, C.; Yang, B.; Liao, X. Binding behavior, water solubility and in vitro cytotoxicity of inclusion complexes between ursolic acid and amino-appended β-cyclodextrins. *J. Mol. Liq.* **2019**, *296*, 111993. doi: 10.1016/j.molliq.2019.111993.
